# Reducing the tendency for chronometric counting in duration discrimination tasks

**DOI:** 10.3758/s13414-022-02523-1

**Published:** 2022-06-14

**Authors:** Martin Riemer, Paula Vieweg, Hedderik van Rijn, Thomas Wolbers

**Affiliations:** 1 Biological Psychology and Neuroergonomics, Technical University Berlin, Fasanenstr. 1, 10623 Berlin, Germany; 2 Aging & Cognition Research Group, German Center for Neurodegenerative Diseases (DZNE), Magdeburg, Germany; 3 Center for Behavioral Brain Sciences (CBBS), Magdeburg, Germany; 4 Department of Experimental Psychology, University of Groningen, Groningen, the Netherlands

**Keywords:** Chronometric counting, Time discrimination, Time perception, Duration discrimination

## Abstract

Chronometric counting is a prevalent issue in the study of human time perception as it reduces the construct validity of tasks and can conceal existing timing deficits. Several methods have been proposed to prevent counting strategies, but the factors promoting those strategies in specific tasks are largely uninvestigated. Here, we modified a classical two-interval duration discrimination task in two aspects that could affect the tendency to apply counting strategies. We removed the pause between the two intervals and changed the task instructions: Participants decided whether a short event occurred in the first or in the second half of a reference duration. In Experiment 1, both classical and modified task versions were performed under timing conditions, in which participants were asked not to count, and counting conditions, in which counting was explicitly instructed. The task modifications led to (i) a general decrease in judgment precision, (ii) a shift of the point of subjective equality, and (iii) a counting-related increase in reaction times, suggesting enhanced cognitive effort of counting during the modified task version. Precision in the two task versions was not differently affected by instructed counting. Experiment 2 demonstrates that—in the absence of any counting-related instructions—participants are less likely to engage in spontaneous counting in the modified task version. These results enhance our understanding of the two-interval duration discrimination task and demonstrate that the modifications tested here—although they do not significantly reduce the effectiveness of instructed counting—can diminish the spontaneous tendency to adopt counting strategies.

An important method in the study of human time perception are psychophysical tasks in which participants make comparative judgments about the duration of acoustic or visual stimuli (Allan, 1979; Grondin, 2008; Riemer, 2015). However, when supra-second intervals need to be judged, participants often resort to chronometric counting strategies (Grondin et al., 1999; Hinton et al., 2004). As these methods affect performance independently from the underlying timing mechanisms, they can contaminate the construct validity of time perception tasks. Enhanced task performance due to chronometric counting reflects the ability to regularly produce very short intervals (i.e., the duration between two subsequent numbers), rather than the perception of elapsed time in the supra-second range. Importantly, many studies suggest that the perception of intervals in the subsecond range is conceptually different from the perception of longer intervals and might resort on different mechanisms (Kagerer et al., 2002; Ulbrich et al., 2007; Zélanti & Droit-Volet, 2011).

One example for which reducing the effectiveness (and hence the use) of compensatory strategies such as chronometric counting is especially important, is the investigation of timing deficits in advanced age (e.g., Maaß et al., 2022; Riemer et al., 2021) and neurodegenerative diseases (e.g., Maaß et al., 2019; Mioni et al., 2021), because older participants and patients with beginning dementia can be very reluctant to admit perceptual impairments. To conceal existing deficits, these groups might rely stronger on compensatory strategies and heuristics to increase their performance. In the domain of spatial navigation, Wiener et al. (2013) reported an age-related bias towards simple but less flexible strategies. Older participants showed a deficit in creating a cognitive map of a new environment and compensated for this deficit by using a relatively easy stimulus–response strategy. In many everyday navigation tasks (e.g., retracing a known route), these participants performed well despite of a significant impairment in their spatial representation of environments. With respect to time perception, Perbal et al. (2002) demonstrated that older adults performed equal to younger adults in a timing task when they were allowed to count, although significant age-related differences were observed when participants were distracted from chronometric counting. These examples demonstrate that core deficits in a specific domain can be obscured by the application of compensatory strategies that are different from the targeted cognitive domain. With respect to the study of human time perception, this highlights the importance of reducing both the effectiveness and the occurrence of chronometric counting in psychophysical tasks.

To prevent chronometric counting, three main techniques have been employed: Concurrent distracter tasks, articulatory suppression and the instruction not to count (for a comparison of these techniques, see Rattat & Droit-Volet, 2012). However, all of these methods are associated with a number of disadvantages. The administration of a concurrent distracter task can be quite efficient to impede chronometric counting (e.g., Brocas et al., 2018; Perbal et al., 2002; Wearden et al., 1997; Wittmann et al., 2010), but it also distracts the attention from the timing process itself. As time perception is strongly influenced by the attentional resources available (Block et al., 2010; Brown, 1997), a distracter task—included for the sake of a pure measure of timing abilities—might itself alter this measure (Hemmes et al., 2004; Rattat & Droit-Volet, 2012). A second technique to prevent chronometric counting is articulatory suppression, that is, the participants are asked to produce irrelevant speech syllables (“bla-bla-bla . . .”) in order to prevent internal vocalization required for counting (e.g., Baudouin et al., 2006; Clément & Droit-Volet, 2006; Droit-Volet et al., 2003; Franssen et al., 2006). Though this method is much less demanding for attention, the discrete regular elements might be counted themselves (e.g., by visually imagining the respective numbers). On all accounts, they accumulate to specific quantities and subsequent judgments of elapsed time might be influenced by these quantities (Dormal et al., 2006; Javadi & Aichelburg, 2012). Furthermore, for studies involving fMRI, EEG and MEG, articulatory suppression would cause muscle artifacts and reduce data quality. A third technique to prevent chronometric counting is simply to instruct the participants not to count (e.g., Akdoğan & Balcı, 2017; Hinton et al., 2004; Riemer et al., 2012; Riemer & Wolbers, 2020). This technique critically depends on the assumption that participants are both capable and willing to adhere to this instruction, an assumption that can be questioned, especially because researchers and participants can have a divergent understanding of counting strategies (e.g., whether it includes attending to one’s own breathing rhythm or imagining a melody). Moreover, if participants are particularly motivated to demonstrate good performance, as might be the case for participants undergoing medical examinations, the mere instruction not to count is not sufficient. Another disadvantage of explicit no-counting instructions is that the active inhibition of an intuitive strategy can itself deplete cognitive resources and distract the participant's attention from the timing process (just as a distracter task). Note that this is an alternative interpretation of the results from Perbal et al. (2002).

In summary, all three techniques have a number of potentially crucial disadvantages. Therefore, it is pertinent to improve on the timing tasks we have at hand by reducing the influence of chronometric counting, and ultimately diminishing the spontaneous tendency to adopt counting strategies. Are there certain aspects in the paradigms we use that could be changed in order to reduce the susceptibility to chronometric counting?

Conventionally used time perception tasks often require a comparison between two temporal intervals (cf. Allan, 1979; Riemer, 2015). In a two-interval duration discrimination task, participants decide whether a comparison interval was shorter or longer than a standard interval (e.g., Grondin, 1993; Riemer et al., 2016). The explicit instruction to *compare the duration* of two intervals might point towards chronometric counting as a potentially useful strategy, because duration differences are often expressed in numerical terms. Furthermore, the short pause between the two intervals (usually about 1 second) enables the memorization of the reached number and leaves enough time to reset and count from zero for the second interval.

In the present study, we modified these two aspects in an attempt to reduce the effectiveness and the application of counting strategies. A graphic depiction of these modifications is shown in Fig. 1. The pause was removed, so that both intervals merged together to one longer interval (in the following referred to as *reference duration*), and instead of the pause a short (40 ms) acoustic beep signal was presented. Participants then indicated whether the acoustic event occurred in the first or in the second half of the reference duration (two-alternative forced choice). In each trial, the length of the reference duration was variable.^1^ and the auditory event was systematically scattered around its midpoint.

**Fig. 1:**
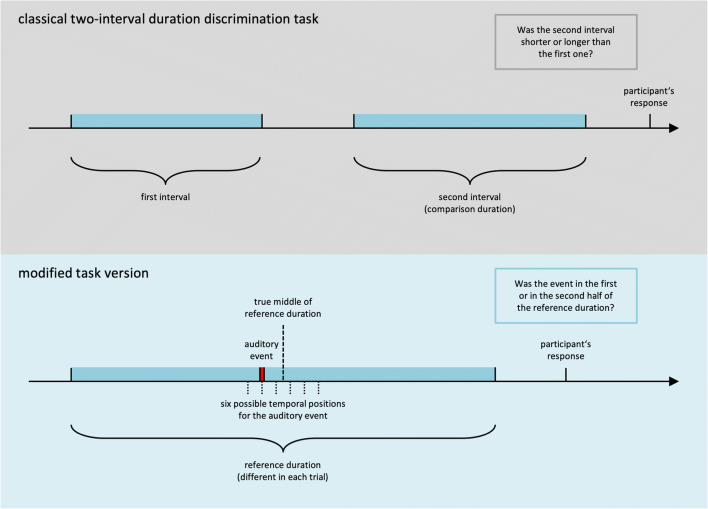
Schematic of the classical two-interval duration discrimination task, in direct comparison with the tested modifications. In the modified task version, the participant has to indicate whether an auditory event occurred in the first or in the second half of the reference duration (in contrast to whether a comparison duration was longer or shorter than a previously presented duration)

In contrast to the classical task version, in which the participants decide whether a comparison duration is shorter or longer than a previously presented duration (Grondin, 1993; Rammsayer & Lima, 1991), the changed instruction explicitly requires the localization of an event *in time* rather than a comparison between two durations. Although this decision also depends on a two-interval comparison, the instruction to *localize an event in time* does not directly point to a comparative strategy. We hypothesized that these differences (i.e., the removal of the pause and the changed instruction) reduce the effect of chronometric counting on judgment precision because (i) the absence of a pause makes it difficult to reset and start counting from zero for the second interval, while at the same time memorizing the reached number for the first interval, and (ii) the instruction to localize an event in time does not directly hint towards a comparative strategy and hence towards a potential benefit of chronometric counting. We had no specific hypotheses regarding judgment accuracy and reaction times.

## Experiment 1

In Experiment 1, the modified version was directly compared against the classical two-interval duration discrimination task. We specifically addressed the question to what extent the performance is influenced by explicitly instructed chronometric counting, relative to a “no-counting” instruction.

### Methods

#### Participants

Fifty-two participants were recruited from the Technical University of Berlin and the local community and equally assigned to either the classical two-interval duration discrimination task (17 females, nine males; mean age 27.8 years, ranging from 20 to 40) or the modified task version (16 females, 10 males; mean age 27.6 years, ranging from 19 to 36). All participants gave written informed consent to the experimental protocol, which was approved by the local ethics committee.

#### Tasks and stimuli

In the modified task version, the reference duration was signalized by a 250 Hz sound (filled duration) and the beep signal by a 2 kHz sound of 40 ms duration. Six different reference durations were used (2.44, 3.05, 3.81, 4.76, 5.95, and 7.44 s). The beep was presented at one of six possible positions during the reference duration, defined in percentage values (40, 44, 48, 52, 56, and 60%). The duration of the beep signal (40 ms) was accounted for by setting its midpoint (not its onset) to the specified temporal position within the reference duration. In sum, six reference durations combined with six relative beep positions were presented in a randomized order, and in four separate blocks, resulting in a total of 144 trials per participant and per task. After the offset of the reference sound, participants had to decide whether the beep signal appeared during the first (left button) or the second half (right button) of the reference sound. Responses were given with the two keys at the left and right outermost lower corners of a standard German keyboard.

The same intervals were used in the classical task version. With respect to the presented stimuli, the only difference between the two versions consisted in the substitution of the pause (1 s) for the beep signal (40 ms). Thus, in the classical task version, participants were presented with a first sound (250 Hz), a short pause (1 s), a second sound (250 Hz), and then they had to decide whether the second sound was shorter (left button) or longer (right button) than the first sound (cf. Fig. 1).

Acoustic stimuli were sine wave sounds delivered binaurally via headphones (Stereo TW-260A). Participants were instructed to close their eyes and wait until the end of the respective sound before giving a response. Each participant performed either the classical or the modified task version under two experimental conditions. No feedback was provided throughout the whole experiment.

#### Experimental conditions and self-ratings

Two experimental conditions were realized. In the timing condition, participants were instructed to refrain from chronometric counting “in order to provide a measure of pure timing performance,” while in the counting condition, they were explicitly instructed to mentally count “in order to optimize timing performance.” The order of experimental conditions (timing vs. counting) was counterbalanced across subjects.

Directly after the completion of each condition (counting and timing), participants were asked to estimate “how often they had given the correct response.” These metacognitive performance estimates were given on a visual analogue scale, the limits of which were labeled as “always guessed” and “always correct.” In the counting condition, participants were then asked how easy it was to apply a counting strategy (“easy” to “difficult”), and in the timing condition, they were asked how easy it was to refrain from counting (“easy” to “difficult”). Finally, after the completion of both conditions, participants rated how much they did benefit from counting, relative to the timing condition (“not at all” to “very much”).

#### Statistical analysis

Responses later than 10 s after the offset of the reference sound (or the second sound) were discarded as outliers (0.4%). For each participant and each experimental condition, psychometrical functions were calculated using R package *quickpsy* (Linares & López-Moliner, 2016). Guess and lapse rates were allowed to vary between 0 and 0.2. Fitted logistic functions represent the probability of the response “beep was in the second half” or “second interval was longer than first” as a function of the relative position of the beep signal or the pause, respectively. Mean accuracy and precision of temporal judgments were quantified by the point of subjective equality (PSE), defined as the value of the *x*-axis corresponding to 50% on the *y*-axis, and the difference limen (DL), defined as half the distance between the values of the *x*-axis corresponding to 25% and 75% on the *y*-axis (Ulrich & Vorberg, 2009). Cases in which the estimated PSE lay outside the tested range of beep/pause positions (i.e., below 0.4 or above 0.6), as well as cases in which the DL deviated more than 2 times the interquartile range from the median (indicating poor performance) were defined as outliers and discarded from further analysis (4.8%). Goodness-of-fit for the psychometric functions was calculated by deviance, ranging from 0.2 to 11.1 (Wichmann & Hill, 2001).

For the analysis of reaction times, responses earlier than 100 ms or deviating more than three standard deviations from the individual mean of the specific task were discarded (1.8%).

Data were analyzed in R (R Core Team, 2016), by fitting linear mixed effects models (2 × 2 factorial design) using packages *lme4* (Bates et al., 2015) and *lmerTest* (Kuznetsova et al., 2017), including the between-subjects factor task version (classical vs. modified, coded as −.5 and .5) and the within-subjects factor condition (timing vs. counting, coded as −.5 and .5). Subjects were included as random factor. A complete analysis script and the raw data for Experiment 1 can be found at OSF (https://osf.io/4rgth/).

### Results

#### Precision

**Fig. 2:**
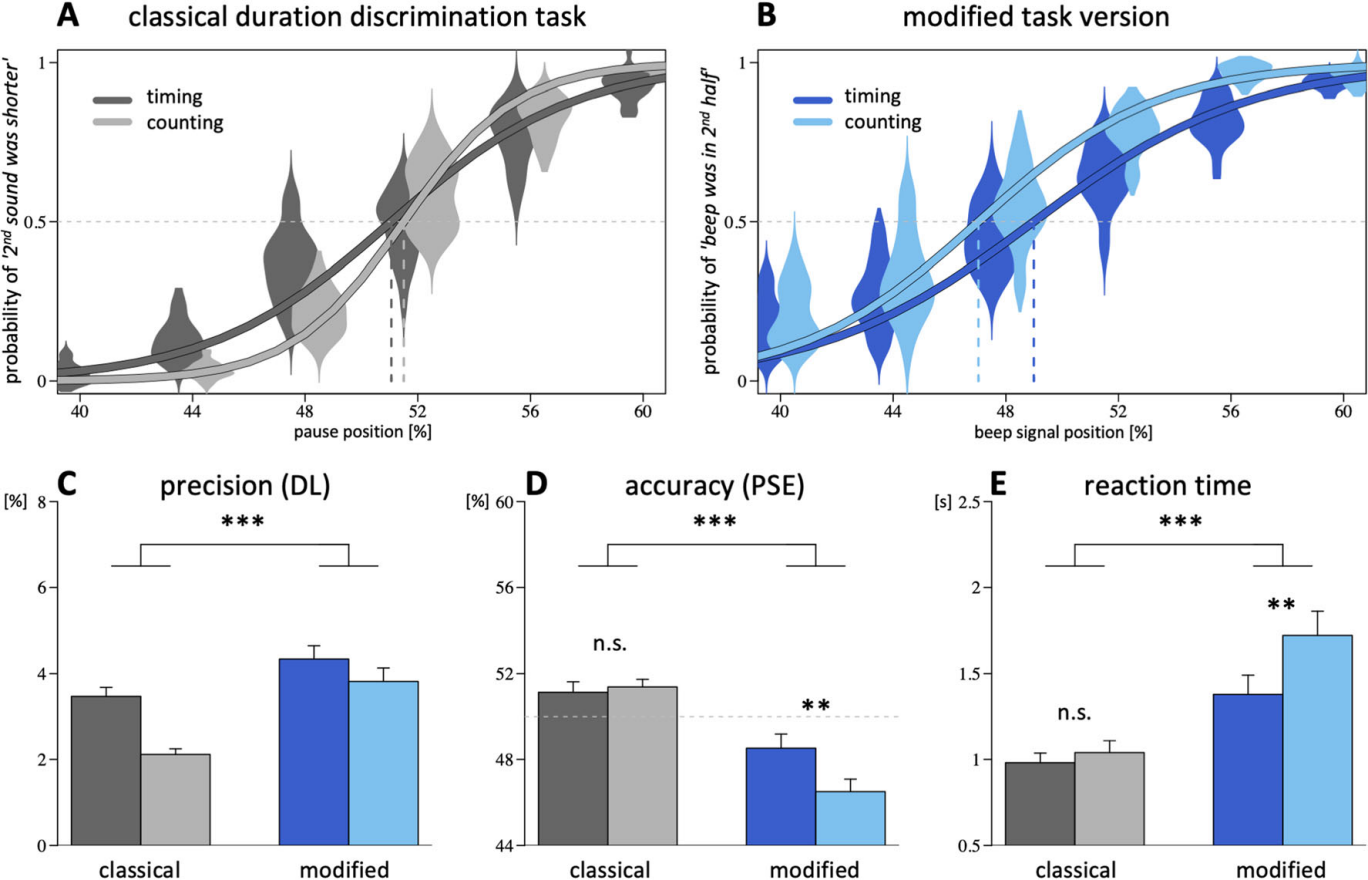
Results from Experiment 1. **a–b** Logistic functions. **c** Precision (smaller DL values indicate higher precision). **d** Accuracy (PSE values close to 50% indicate higher accuracy). **e** Reaction times for the classical (grey) and the modified task version (blue), under timing (dark shades) and counting conditions (light shades). Vertical dashed lines indicate the point of subjective equality. Error bars show standard error across subjects. ***p < .001. **p < .01. ^n.s.^*p* > .10

Results are depicted in Fig. 2. A main effect of task version indicates a higher precision in the classical as compared with the modified version, β = 0.013, *SE* = 0.003, *t*(50) = 5.0, *p* < .001, and a main effect of condition indicates a higher precision for the counting as compared with the timing condition, β = −0.009, *SE* = 0.002, *t*(50) = −3.9, *p* < .001. No significant interaction between condition and task version was found, β = 0.008, *SE* = 0.005, *t*(50) = 1.8, *p* = .082.^2^

#### Accuracy

Regarding judgment accuracy (Fig. 2c), the main effects indicate a different PSE bias between the task versions, β = −0.038, *SE* = 0.006, *t*(48) = −6.1, *p* < .001, and between conditions, β = −0.009, *SE* = 0.004, *t*(46) = −2.2, *p* = .034. We also found a significant interaction between task version and condition, β = −0.022, *SE* = 0.008, *t*(46) = −2.8, *p* = .007. Subsequent two-tailed t-tests revealed that the effect of counting was entirely due to the modified task version, *t*(20) = 2.9, *p* = .008, while the classical task version was not affected, *t*(25) = −0.5, *p* > .5.

Two-tailed t-tests against the point of objective equality, separately conducted for each task version, showed that the PSE between first and second interval in the classical version was biased towards larger values—timing: *t*(25) = 2.3, *p* = .029; counting: *t*(25) = 3.9, *p* < .001—while the analogous PSE between the interval’s first and second half in the modified version was biased towards smaller values—timing: *t*(21) = −2.2, *p* = .038; counting: *t*(24) = −6.0, *p* < .001.

#### Reaction times

The analysis of reaction times (Fig. 2d) revealed significant main effects for task version, β = 0.541, *SE* = 0.129, *t*(50) = 4.2, *p* < .001, and condition, β = 0.201, *SE* = 0.056, *t*(50) = 3.6, *p* < .001, as well as for their interaction, β = 0.284, *SE* = 0.111, *t*(50) = 2.6, *p* = .013. Further analysis of this interaction by means of two-tailed *t* tests demonstrated that the effect of instructed counting on reaction times is entirely based on differences in the modified version, *t*(25) = 3.4, *p* = .002, while reaction times in the classical task version were not significantly different between the timing and counting condition, *t*(25) = 1.2, *p* = .23. This finding is in line with the assumption that chronometric counting increases the cognitive load in the modified task version significantly more than in the classical task version.

#### Performance self-ratings

**Fig. 3:**
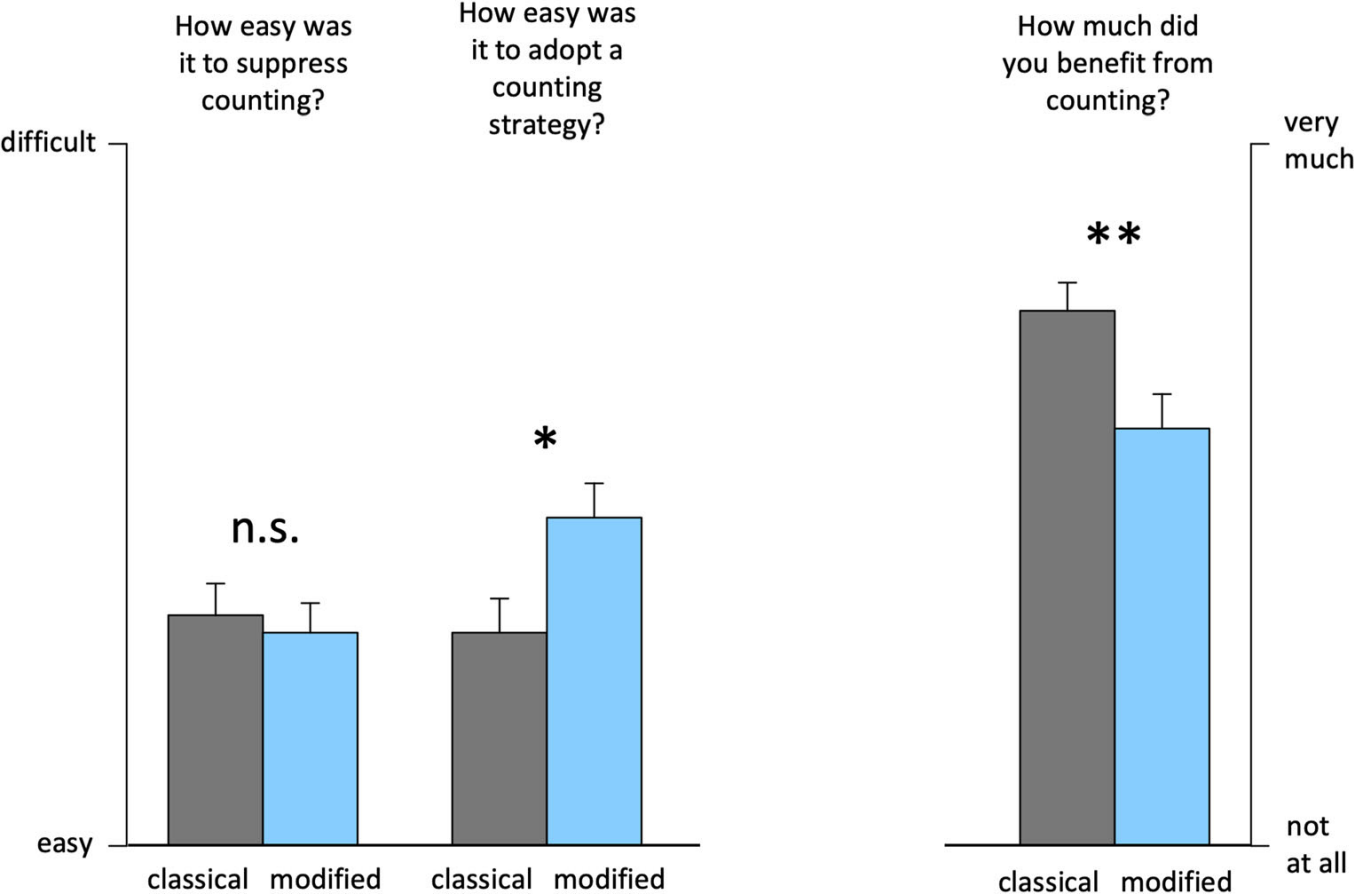
Retrospective self-reports for Experiment 1. Difficulty to suppress counting, difficulty to adopt a counting strategy, and perceived benefit of chronometric counting for the classical (grey bars) and the modified task version (blue bars). **p < .01. *p < .05. ^n.s.^*p* > .10

*T* tests on self-ratings revealed no significant difference in the perceived level of performance between the two task versions, neither after the timing condition, |*t*(47)| < 0.1, *p* > .5 (two-tailed) nor for the counting condition, *t*(48) = 1.5, *p* = .15 (two-tailed). Subjective ratings about the difficulty of counting (or of suppressing it, respectively) and of the general benefit of counting are shown in Fig. 3. Chronometric counting was rated as easier during the classical as compared with the modified task version, *t*(50) = 2.4, *p* = .011 (one-tailed), while no such difference between the task versions was found when the difficulty to suppress counting was rated, *t*(50) = −0.4, *p* = .34 (one-tailed). Finally, we asked the participants how much they felt having benefitted from counting (relative to the timing condition). This perceived benefit of chronometric counting was larger in the classical compared with the modified task version, *t*(48) = 2.6, *p* = .006 (one-tailed).

### Discussion

Many studies using a variety of psychophysical methods have shown that chronometric counting results in much more precise temporal judgments compared with a no-counting instruction (Gaudreault & Fortin, 2013; Grondin et al., 1999; Hinton et al., 2004; Rakitin et al., 1998; Wearden et al., 1997). In line with those studies, the results from the classical two-interval duration discrimination task confirmed that our participants were able to use chronometric counting for the tested durations and that they benefitted from employing a counting strategy. Contrary to our hypothesis, this counting-related performance advantage was still present in the modified version of the task, in which we removed the pause and changed task instructions. However, retrospective self-reports also showed that the perceived benefit of chronometric counting was larger in the classical than in the modified task version. Furthermore, specifically in the modified task version, participants reported greater difficulties of adopting a counting strategy.

Thus, even though the two modifications of the two-interval duration discrimination task do not make it resistant to the facilitating effect of chronometric counting, they might impede the spontaneous use of counting strategies due to an increased difficulty of employing such strategies. This interpretation is supported by the results regarding reaction times. Specifically in the modified task version, counting resulted in higher reaction times, which could be indicative of increased cognitive load.

Even though the applied task modifications do not reduce the effectiveness of chronometric counting itself, it might reduce the spontaneous tendency to use counting as a compensatory strategy—that is, when no explicit counting-related instructions had been given. If the participants follow a timing strategy (as researchers usually want them to), there is no difference in the perceived difficulty to suppress counting. Only if participants deliberately engage in chronometric counting do they perceive this as being more difficult during the modified task version.

In Experiment 2, we therefore investigated the idea that the spontaneous tendency to adopt a counting strategy can be reduced by modifications of the classical two-interval duration discrimination task.

## Experiment 2

Experiment 2 was conducted to investigate the tendency to spontaneously engage in chronometric counting in the absence of any counting-related instructions. If the modified task version reduces the intuitiveness of the idea that counting might be a helpful strategy, participants should automatically engage less in chronometric counting than they do in the classical two-interval duration discrimination task.

### Methods

#### Participants and experimental groups

Experiment 2 was conducted at the University of Groningen. Eighty-nine German-speaking participants were recruited and randomly assigned to one of two groups, performing either the classical task version (26 females, 19 males; mean age 20.4 years, ranging from 18 to 29) or the modified version (27 females, 17 males; mean age 20.6 years, ranging from 18 to 26). Importantly, only first-year students who did not previously participate in similar timing experiments were included in Experiment 2. This ensured that all participants were unfamiliar with the usual instruction to refrain from chronometric counting, which is used in many timing studies. All verbal and written instructions were given in German. All participants gave written informed consent to the experimental protocol, which was approved by the local ethics committee.

#### Task, stimuli, and self-ratings

Stimulus material was the same as in Experiment 1. We compared the performance in the classical and the modified task version, in the absence of any explicit instructions regarding chronometric counting. A between-subjects design was realized, in order to gain a pure estimation of the counting frequency during the specific task version.

As the primary focus of Experiment 2 was the analysis of self-reports, the trial number for each task version was reduced to 36 trials (comparable to one block in Experiment 1). Each participant performed one task version (classical or modified). After completion of the task, participants were informed that “people sometimes find this task easier to solve when using a mental counting strategy,” and they were asked to indicate on a visual analogue scale, in how much percentage of all trials, they did that themselves. They also rated how much this counting strategy (if adopted) had improved their performance. Finally, participants were asked whether they had—during the experiment—any assumptions as to whether they were expected to refrain from counting or not. All other aspects were equal to Experiment 1.

#### Statistical analysis

Responses later than 10 s were discarded as outliers (0.1% of all trials). Psychometrical functions were calculated as described in Experiment 1. Goodness-of-fit parameter deviance ranged from 0.1 to 16.1 (Wichmann & Hill, 2001). Outliers were defined by the same cutoff criteria specified in Experiment 1 and discarded from further analysis (9.0%). For the analysis of reaction times, 1.5% of trials were discarded according to the criteria specified in the methods section of Experiment 1. All data were analyzed with t-test for unpaired samples.

For the analysis of retrospective self-reports regarding the spontaneous engagement in chronometric counting, we excluded participants who refrained from counting due to a previously existing assumption that exactly this was expected from them (10.1% of all participants).

As no counting-related instructions were provided in Experiment 2, it was not possible to compare the performance between counting and timing conditions. However, we analyzed the correlation coefficients between task performance and self-reported spontaneous counting, which were calculated using R package MASS for robust linear models (Venables & Ripley, 2002). Direct comparison between correlation coefficient was done with function *paired.r* of R package *psych*. A complete analysis script and the raw data for Experiment 2 can be found at OSF (https://osf.io/4rgth/).

### Results

#### Performance self-ratings

**Fig. 4:**
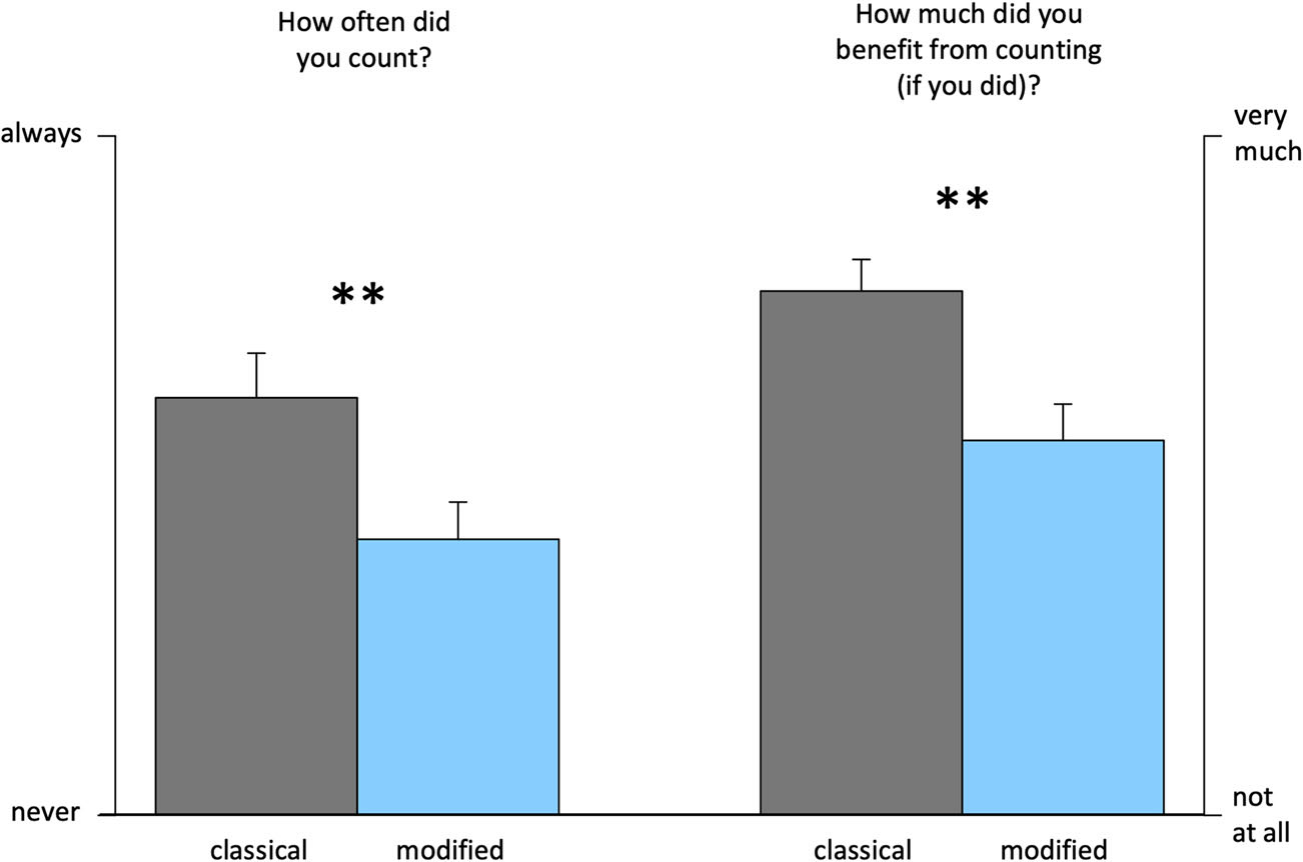
Ratings of spontaneous chronometric counting and perceived benefit of counting in Experiment 2. Spontaneous counting occurred significantly less during the modified (blue) as compared with the classical two-interval duration discrimination task (grey). **p < .01. (Color figure online)

The main focus of Experiment 2 was the question about the spontaneous engagement in chronometric counting (Fig. 4). The analysis of retrospective self-reports revealed that participants engaged significantly less in chronometric counting during the modified task version, *t*(74) = 2.4, *p* = .009. Moreover, the benefit of counting was judged as lower in the modified as compared with the classical task version, *t*(59) = 3.1, *p* = .002.

#### Precision, accuracy and reaction times

**Fig. 5:**
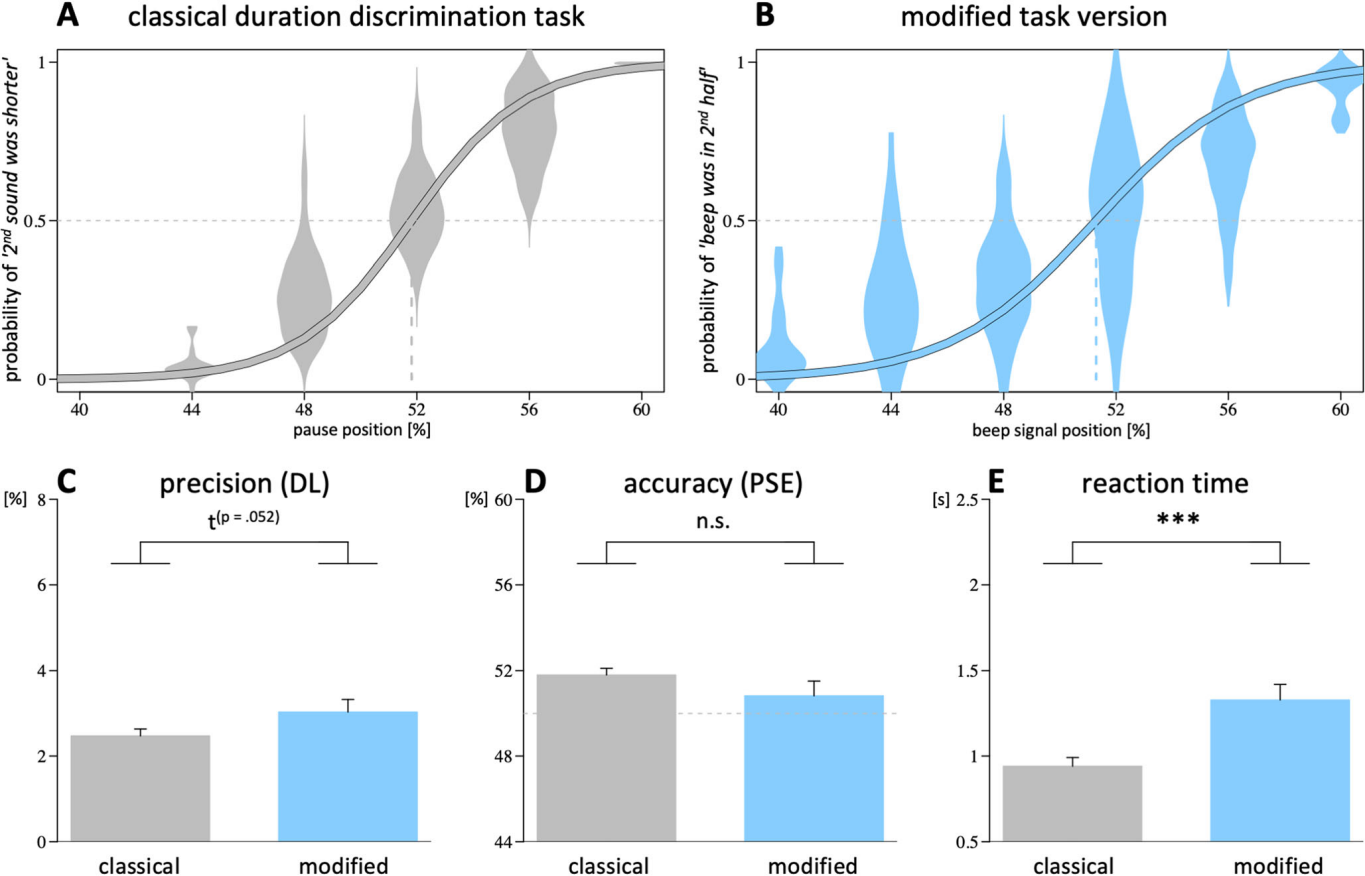
Results from Experiment 2. **a–b** Logistic functions. **c** Precision (smaller DL values indicate higher precision). **d** Accuracy (PSE values close to 50% indicate higher accuracy). **e** Reaction times for the classical (grey) and the modified task version (blue). Vertical dashed lines indicate the point of subjective equality. Error bars show standard error across subjects. ***p < .001; ^n.s.^*p* > .10. (Color figure online)

Results are depicted in Fig. 5, analogous to the presentation of results from Experiments 1. Precision was higher in the classical as compared with the modified task version, *t*(55) = 1.7, *p* = .052 (one-tailed). The PSE was biased towards larger values in the classical task version, *t*(44) = 5.7, *p* < .001 (two-tailed), while it was not in the modified task version, *t*(35) = 1.2, *p* = .24. However, the PSE difference between the task versions did not reach statistical significance, *t*(49) = 1.3, *p* = .20 (two-tailed). Again, reaction times were significantly higher during the modified version, *t*(67) = 3.7, *p* < .001.

**Fig. 6:**
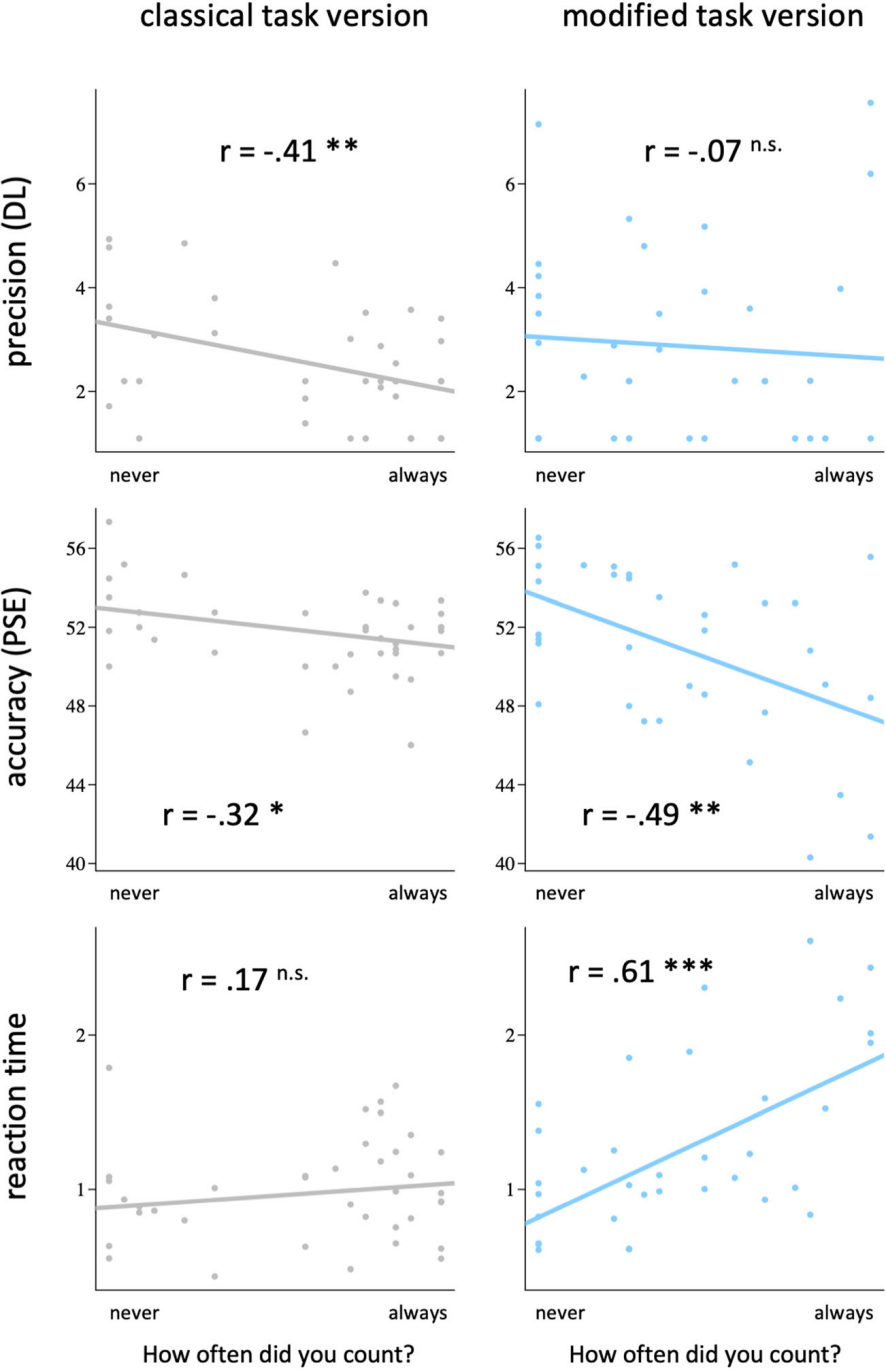
Results from correlation analyses of Experiment 2. The tendency to adopt a counting strategy correlated positively with precision in the classical, but not the modified task version (note that lower DLs indicate higher precision). ***p < .001. **p < .01. *p < .05. ^n.s.^*p* > .10

Results from the correlation analyses are shown in Fig. 6. In the classical task version, the tendency to adopt a counting strategy correlated negatively with the difference limen, *t*(36) = −2.5, *p* = .008, *r* = −.41, that is, the more the participants engaged in chronometric counting, the more precise they were in their judgments. This correlation was absent in the modified version, *t*(32) = −0.5, *p* = .33, *r* = −.07, indicating that the tendency to count—in addition to being less present in general—had no effect on precision. However, a direct comparison of the two correlation coefficients showed no significant difference between the two task versions (*p* = .07; one-tailed).

With respect to accuracy, we found a significant negative correlation of the tendency to count with the PSE for both task versions—that is, the more the participants engaged in chronometric counting, the more their judgments were biased towards lower values, classical: *t*(36) = −2.3, *p* = .014, *r* = −.32; modified: *t*(32) = −3.1, *p* = .002, *r* = −.49. Note that for both task versions, this denotes an attenuated positive bias (i.e., higher accuracy). The correlation coefficients were not significantly different (*p* = .39; two-tailed).

The tendency to count was positively correlated with reaction times in the modified, *t*(32) = 3.6, *p* < .001, *r* = .61, but not in the classical task version, *t*(36) = 1.0, *p* = .17, *r* = .17. This is in line with the results from Experiment 1, suggesting that chronometric counting imposes additional cognitive load in the modified, but not the classical task version. The correlation coefficients differed significantly from each other (*p* = .02; one-tailed).^3^

### Discussion

The results of Experiment 2 demonstrate that participants, who do not hold a specific assumption as to whether chronometric counting is allowed in timing experiments, and also do not receive any explicit instruction regarding counting, exhibit a smaller tendency to count when the classical two-interval duration discrimination task is modified by removing the pause and changing task instructions. Thus, even though in Experiment 1 a reduction of the precision-increasing effect of chronometric counting could not be verified, the results of Experiment 2 suggest that counting strategies are less intuitive in the modified task version.

Furthermore, analyses of the correlation between task performance and self-reports revealed a link between spontaneous counting and precision for the classical but not for the modified task version. With respect to the classical task version, this result mirrors the strong precision-increasing effect of chronometric counting found in Experiment 1 (cf. Fig. 3b). With respect to the modified task version, the pattern of results is less consistent. In Experiment 2, higher engagement in counting during the modified task version was not related to higher precision.

## General discussion

The present paper investigates the effect of chronometric counting on a two-interval duration discrimination task, a well-established paradigm in time perception research. We modified this classical task in two critical aspects—namely, by (i) removing the pause between the two successive intervals and by (ii) changing the task instructions. In the modified task version, participants are presented with a variable reference duration and an acoustic event approximately in the middle of this reference duration. In each trial, participants have to decide whether the event occurred in the first or in the second half of the reference duration (cf. Fig. 1). In order to determine how these modifications affect the influence of chronometric counting, the performance in the classical and the modified task version was compared between a timing condition (in which counting was not allowed) and a counting condition (in which counting was explicitly instructed). In a second experiment, we compared both task versions with respect to the participants’ tendency to spontaneously engage in a counting strategy, when no explicit counting-related instructions were provided.

As chronometric counting can artificially enhance the performance in timing tasks and conceal underlying deficits, the investigation of single constituents of often-used time perception tasks is an important endeavor and promotes a more efficient application of these tasks. In separate sections, we will discuss how the task modifications affect the influence of instructed counting on the precision of judgments, on the perceived benefit of counting, on judgment accuracy, and on reaction times.

### Precision

In Experiment 1, we did not find a significant difference between the classical and the modified task version with respect to how much the precision of judgments is influenced by chronometric counting. In both tasks, precision was higher when participants were instructed to employ a counting strategy. Similarly, correlation analyses in Experiment 2 revealed no significant difference between the two tasks. Although the correlation between self-reported spontaneous counting and precision was higher in the classical compared with the modified task version (cf. upper graphs in Fig. 6), the difference between the correlation coefficients was only significant by trend. The idea that the modifications of the classical discrimination task reduced the precision-increasing effect of chronometric counting could statistically not be confirmed. In addition, it has to be considered that in Experiment 2, no explicit instructions as to whether counting was allowed or not were provided, and the uncertainty regarding this matter might have increased the intrasubject variability of employed strategies during the task. For example, participants might have started without counting and decided to use a counting strategy later. Ultimately, the increased variability of strategies could have reduced the chance to find a correlation between self-reported counting and precision of judgments in the modified task version. The results of both experiments also reveal a generally lower precision for the modified task version, which might have obscured the correlation between spontaneous counting and precision in Experiment 2.

Taken together, we conclude that for both the classical two-interval discrimination task and the modified version of this task, precision is increased by explicitly instructed chronometric counting. However, in both experiments the perceived benefit of counting was rated higher during the classical task version, which leads to the idea that removing the pause and changing task instructions renders chronometric counting a less intuitive strategy (and therefore less likely to be used).

### Perceived benefit of counting

As the tendency to spontaneously engage in a counting strategy (when no counting-related instructions are provided) largely depends on the participants’ intuition as to whether such a strategy is helpful or not, the analysis of the participants’ subjective experience is of particular significance here. In both experiments, the retrospective self-reports reveal a consistent pattern with respect to the perceived benefit and difficulty of chronometric counting during the classical and the modified task version (Figs. 3 and 4). Participants consistently felt they benefitted more from counting during the classical than during the modified task version. Furthermore, while the difficulty to refrain from counting was not perceived as different between the two versions, participants reported relatively more problems to adopt a counting strategy in the modified as compared with the classical task version (Fig. 3). This pattern suggests that both task versions are comparable with respect to the perceived difficulty to comply with a no-counting instruction, but that, when participants deliberately try to engage in chronometric counting, the task modifications implemented here impede this strategy. Critically, the decrease in the perceived benefit of counting and the increase in perceived difficulty to adopt a counting strategy resulted in a reduced tendency to spontaneously engage in counting (Fig. 4).

Retrospective reports on the perceived benefit of counting are also interesting with respect to the process of temporal error monitoring (Akdoğan & Balcı, 2017; Öztel et al., 2021; Riemer et al., 2019). According to the retrospective self-reports, the advantage of chronometric counting was clearly perceived during the classical discrimination task, while participants seemed to be much less aware of it during the modified task version. We argue that this reduced awareness of the adjuvant effect of counting is the reason why participants were less prone to engage in a counting strategy.

### Accuracy

In both experiments we consistently found a positive bias of the PSE in the classical task version. This reflects the well-known phenomenon of order effects, that is, the tendency to judge the second interval as longer than the first interval (Fechner, 1860; Hellström, 2003; Hellström & Rammsayer, 2004, 2015; Jamieson & Petrusic, 1975; Ulrich & Vorberg, 2009). Interestingly, the modifications of the classical task reduced this bias, leading even to a reversed bias in Experiment 1. It therefore seems that the temporal order effect in the classical task version critically depends on the presence of a pause (and its duration) between the first and the second interval (Hellström, 2003; Hellström & Rammsayer, 2004).

An attenuation of the PSE might also reflect the use of a different cognitive mechanism underlying performance in the modified task version. For example, one could speculate that, in the modified task version, the stimuli are perceived as a series of three events (reference onset–beep signal–reference offset)^4^ and processed with respect to their rhythmic qualities (e.g., Rhodes & Di Luca, 2016). In future studies, this hypothesis could be tested by implementing shorter reference durations, because the perception of rhythm is restricted to a fast succession of auditory stimuli and most studies on rhythm perception focus on interstimulus intervals in the range of milliseconds (e.g., Povel, 1984; Rhodes & Di Luca, 2016). However, it is important to keep in mind that the use of shorter durations itself (i.e., independent of the task) might prompt different timing strategies, as many studies support the view that the processing of short and long durations is based on different mechanisms (Fraisse, 1984; Kagerer et al., 2002; Ulbrich et al., 2007; Zélanti & Droit-Volet, 2011).

Another interesting aspect regarding the modified task version is the finding that instructed counting induced a change in the PSE bias. This finding contrasts with the usual observation of either equal (Clément & Droit-Volet, 2006; Hinton et al., 2004) or increased timing accuracy under conditions of instructed counting (Bartholomew et al., 2015; Gaudreault & Fortin, 2013; Grondin et al., 1999; Rattat & Droit-Volet, 2012). A potential explanation for this effect builds on the fact that the absence of a pause in the modified task version makes it harder to stop counting for the first and, at the same moment, start counting for the second half of the reference interval. Assuming that the participants are overstrained with this effort and start counting for the second half too late, one would expect that they reach a smaller number during the second half and, ultimately, this should result in an increased bias to judge the beep signal as occurring in the second half (or the first interval as longer). According to these considerations, the counting-induced effect on the PSE should only be present in the modified task version, which matches our results.

### Reaction times

Another consistent finding in the present study consists in the counting-related increase of reaction times, which was found selectively for the modified task version (Fig. 2d). In Experiment 1, instructed counting resulted in increased reaction times in the modified, but not in the classical task version, and in Experiment 2, self-reported spontaneous counting was positively correlated with reaction times, again selectively in the modified task version. As reaction times usually increase due to enhanced cognitive task demands, this pattern suggests a selective counting-related increase in cognitive load for the modified task version. Application of a counting strategy seems to be easy and intuitive during the classical task version, while it enhances the cognitive demands in the modified version. Together with the self-reports regarding the difficulty of adopting a counting strategy (which was rated higher during the modified task version; cf. Fig. 3), the counting-related increase of reaction times demonstrates the lack of intuitiveness of chronometric counting under the modified task conditions.

### Effects of chronometric counting

There is broad evidence for impaired time perception in many neurodegenerative diseases, like Parkinson’s disease (Gu et al., 2016; Harrington et al., 2011; Merchant et al., 2008) and Alzheimer’s disease (Caselli et al., 2009; El Haj et al., 2013; Rueda & Schmitter-Edgecombe, 2009). Time perception tasks might therefore reveal important information for clinical diagnostics (Maaß et al., 2019; Maaß et al., 2022). However, the value of time perception tasks as a diagnostic tool is substantially reduced by the possibility of chronometric counting as a compensating strategy. When patients can increase their timing performance by applying a compensatory strategy like chronometric counting, it impedes the degree to which clinicians can draw inferences from the task performance to underlying deficits in the cognitive function under investigation. It can even be assumed that patients rely more on compensatory strategies the more they are impaired in the cognitive function of interest. Therefore, it is of greatest importance to investigate the effects of chronometric counting and the factors supporting this strategy in established paradigms that are frequently used in time perception research, as the two-interval duration discrimination task.

The modifications of a classical two-interval duration discrimination task presented in the present study do not prevent the effect of instructed chronometric counting on judgment precision. However, they render counting strategies less intuitive and more difficult to employ, and in the absence of any counting-related instructions, spontaneous engagement in chronometric counting is reduced.

## Conclusion

We investigated the two-interval duration discrimination task by testing the counting-related effects of two modifications to the classical task version: We removed the pause between the successive intervals (substituting it for a short auditory beep signal) and changed the task instructions from “discriminate two durations” to “localize an auditory event in time.”

Direct comparisons between the modified and the classical task version showed that these modifications (i) do not inhibit the precision-increasing effect of chronometric counting, but (ii) reduce the tendency to spontaneously engage in a counting strategy. Furthermore, we found that the task modifications reversed the PSE bias—that is, the tendency to judge the second interval as longer than the first one. A further examination of these and similar modifications of established time perception tasks can provide useful information for future studies, as chronometric counting is a serious obstacle in time perception research (Rattat & Droit-Volet, 2012).

## Footnotes

1 Varying reference durations is essential, because it prevents participants from processing only the first half of the duration (i.e., unitl the beep occurs). If reference durations are identical across trials, participants could adapt to this continguency and base their judgments on the duration of the first half, without attending to the complete reference duration.

2 As many studies have shown that the effect of chronometric counting depends on the length of tested durations (Grondin et al., 1999; Grondin et al., 2004), we ran two additional analyses separately for relatively short versus long reference durations. For both task versions, these analyses revealed a precision-increasing effect of counting for long, main effect of condition: β = −0.013, *SE* = 0.002, *t*(48) = −6.1, *p* < .001; interaction with task version: β = −0.002, *SE* = 0.004, *t*(48) = −0.5, *p* > .5, but not for short durations, main effect of condition: |β| < 0.001, *SE* = 0.002, *t*(47) < .10, *p* > .5; interaction with task version: β = 0.002, *SE* = 0.005, *t*(47) = 0.5, *p* > .5.

3 The difference between the correlation coefficients remained statistically significant (*p* = .042) when tested with a bootstrap method (Rousselet et al., 2021).

4 Using “empty” intervals as reference durations (with only onset and offset of the critical interval being indicated by short sounds; see Grondin, 1993; Rammsayer & Lima, 1991) would render such an interpretation even more likely.
